# Betulin Accelerated the Functional Recovery of Injured Muscle in a Mouse Model of Muscle Contusion

**DOI:** 10.7150/ijms.87649

**Published:** 2024-01-01

**Authors:** Yueh-Hsiu Lu, Yi-Fu Huang, Cheng-Pu Hsieh, Jr-Kai Chen, Hsuan-Ying Chen, Show-Mei Chuang

**Affiliations:** 1 Institute of Biomedical Sciences, National Chung Hsing University, Taichung, 40227, Taiwan.; 2 Department of Orthopedic Surgery, Changhua Christian Hospital, Changhua, 50006, Taiwan.; 3 Orthopedics & Sports Medicine Laboratory, Changhua Christian Hospital, Changhua, 50006, Taiwan.; 4 Department of Post-Baccalaureate Medicine, College of Medicine, National Chung Hsing University, Taichung, 40227, Taiwan.; 5 Department of Kinesiology, Health and Leisure Studies, Chien Kuo Technology University, Changhua, 50094, Taiwan.; 6 Department of Law, National Chung Hsing University, Taichung, 40227, Taiwan.

**Keywords:** betulin, drop-mass injury, functional recovery, muscle contusion

## Abstract

Muscle contusion is an injury to muscle fibers and connective tissues. It commonly happens in impact events, and could result in pain, swelling, and limited range of motion. Diclofenac is one of commonly used nonsteroidal anti-inflammatory drugs to alleviate pain and inflammation after injury. However, it can potentially cause some side effects including gastrointestinal complications and allergy. Betulin is a lupine-type pentacyclic triterpenoid. It is showed to have valuable pharmacological effects, but the physiological effect of betulin on muscle contusion has not been reported. This study aimed to explore the therapeutic effects of betulin on muscle contusion that produced by the drop-mass method in mice. C57BL/6 mice were randomly assigned to control (no injury), only drop-mass injury (Injury), diclofenac treatment (Injury+diclofenac), and betulin treatment (Injury+betulin) groups. Injury was executed on the gastrocnemius of the right hind limb, and then phosphate-buffered saline (PBS), diclofenac, or betulin were oral gavage administrated respectively for 7 days. Results revealed that betulin significantly restored motor functions based on locomotor activity assessments, rota-rod test, and footprints analysis. Betulin also attenuated serum creatine kinase (CK) and lactate dehydrogenase (LDH) levels after muscle injury. Neutrophil infiltration was alleviated and desmin levels were increased after betulin treatment. Our data demonstrated that betulin attenuated muscle damage, alleviated inflammatory response, improved muscle regeneration, and restored motor functions after muscle contusion. Altogether, betulin may be a potential compound to accelerate the repair of injured muscle.

## Introduction

Muscle injuries are the most common injuries with an incidence rate of 10% - 55% during sports activities with contusion as the most common. Muscle contusion is a direct, blunt, compressive force to a muscle that causes muscle or connective tissues injuries. Localized swelling, pain, neutrophil infiltration, edema, and hemorrhage are usually observed in the damaged tissue 1, 2. Inflammation is important for muscle regeneration in the initial stage of injury, but it may cause secondary damage and be detrimental to healing if the response is hyperactive 3, 4.

Present conventional treatment for skeletal muscle injuries includes RICE (rest, ice, compression, and elevation) protocol, intramuscular corticosteroids, immobilization, and nonsteroidal anti-inflammatory drugs (NSAIDs) 5. NSAIDs are regularly ordered as pain relievers and to diminish inflammation. The anti-inflammatory action of NSAIDs is mediated through blocking cyclooxygenase-1 and cyclooxygenase-2 activity, and then reducing arachidonic acid generation to prostaglandin E2 and prostaglandin D2 6. However, problems of foregut symptoms, peptic ulcer, and NSAIDs-induced allergy should be resolved 7. George et al. indicates that NSAIDs administration within 24 h after injury impairs muscle satellite cell proliferation 8.

*Torenia concolor* Lindley var. *formosana* Yamazaki (TC) is a traditional Chinese medicinal herb in Taiwan, and commonly used to dissipate hematoma, reduce inflammation, and alleviate aching muscles after injury in folk medicine 9. Betulin is one of the efficacious components extracted from TC, and it is a lupine-type pentacyclic triterpenoid 10. Betulin has exhibited anti-cancer effects via inducing autophagy and cell cycle arrest and promoting apoptosis in osteosarcoma and colorectal cancer cell lines 11, 12. Betulin is reported to attenuate oxidative stress after ischemia/reperfusion injury 13, and has a strong anti-inflammatory activity via reducing the tumor necrosis factor-α (TNF-α), interleukin-1β (IL-1β) and interleukin-6 (IL-6) levels in damage tissues 14, 15. However, there were fewer studies to explore the physiological effects of betulin on muscle injury.

Since NSAIDs have adverse side effects and excessive blocking on inflammatory responses may result in incomplete healing 6-8, safe and effective medication to treat muscle injury are needed. Betulin has been reported to have valuable pharmacological activities. Thus, present research aimed to explore the potential therapeutic effects of betulin on drop-mass-induced muscle injury.

## Materials and methods

### Animals

Male C57BL/6 mice (8 weeks old; 25-30 g) were obtained from National Laboratory Animal Center (Taipei, Taiwan). The animals were acclimatized to the new environment one week and then randomly assigned into the following groups (6 mice/ group): 1. control group (no injury, PBS-treated, oral gavage), 2. injury group (with drop-mass injury, PBS-treated, oral gavage), 3. injury+ diclofenac group (with drop-mass injury, diclofenac-treated, 10 mg/kg BW, oral gavage), and 4. injury+ betulin group (with drop-mass injury, betulin-treated, 10 mg/kg BW, oral gavage). Diclofenac and betulin powder were purchased from the Cayman Chemical (Ann Arbor, Michigan, USA). All animals were received humane care following the guidelines of the Institutional Animal Care and Use Committee of Changhua Christian Hospital (CCH-AE-110-012). The “resource equation” approach was used to calculate the number of animals 16, 17. Based on this approach, the acceptable range of the error degrees of freedom (DF) in an analysis of variance (ANOVA) is between 10 to 20. DF = Total number of animals - Total number of groups. In this study, DF is 20 that located in the acceptable range.

### Drop-mass-induced muscle injury model

The muscle contusion to the rodent hind limb was induced using the drop-mass method, which was first established by Stratton et al. in 1984 18. We used the method according to a previous study with modification 19, 20. Briefly, a 50 g weight was dropped from a height of 40 cm onto the right gastrocnemius muscle under 4%-5% isoflurane anesthesia. The damage is moderately severe and does not cause bone injury after contusion.

### Open field test

The open field test was performed on 0 day (before injury), 3 and 7 days (after injury) in a dark and quiet room. Mice were put in the center of a behavior box (40 × 40 × 30 cm, length × width × height), and the spontaneous behavior was recorded for 5 min. The videos were evaluated using Ethovision XT version video tracking system (Noldus Information Technology, the Netherlands) to measure the total moving distance (cm).

### Rota-rod test

We used the rota-rod apparatus (YLS-31A, Jinan Yiyan Technology Development Co., Ltd.) to record the latency time that mice remained on a revolving rod to assess motor ability. The test was performed on 0 day (before injury), 3 and 7 days (after injury). The initial speed is 4 rpm, and linearly increases to 30 rpm until 5 min. The latency time was recorded, and a higher latency time indicates better motor ability.

### Footprints analysis

The hind feet of mice were painted with blue ink. The footprints were printed on the paper on the runway floor when the mice walked across a plastic runway in the dark (6 × 6 × 30 cm, width × height × length). Stride length was measured between the second to fourth footprints of the damage side.

### Muscle damage biomarkers assessments

Mice were sacrificed through CO_2_ inhalation at the end of the experiment (7 days after injury). Blood samples were collected through cardiac puncture, and using an automatic analyzer for serum creatine kinase (CK), and lactate dehydrogenase (LDH) analysis (DRI-CHEM NX 500, Fujifilm Corporation, Tokyo, Japan).

### Histopathology and immunohistochemistry (IHC) analysis

Mice were sacrificed through CO_2_ inhalation at the end of the experiment (7 days after injury). Gastrocnemius muscle was fixed in 4% paraformaldehyde, and further for histopathological analysis. Muscle samples were embedded in paraffin and then hematoxylin and eosin, immunohistochemistry staining (anti-desmin, 1:100, ab15200, Abcam, Cambridge, MA) were performed for the histopathological muscle regeneration marker, and Masson's trichrome staining 19 was used for collagen fibers assessment. Images were obtained by a bright field microscopy (Olympus BX 61, Olympus, Japan), and quantitated by Image J software (National Institutes of Health, Bethesda, Maryland, U.S.A.). Quantitative data were presented as means ± S.E.M.

### Statistical Analysis

All results of present study were expressed as means ± standard error of the mean (n = 6). One-way ANOVA of variance and Duncan's post hoc test were used for statistical data analysis. *P*-values of < 0.05 were considered a statistically significant difference.

## Results

### Betulin restores motor functions after drop-mass-induce muscle injury

**Figure 1:**
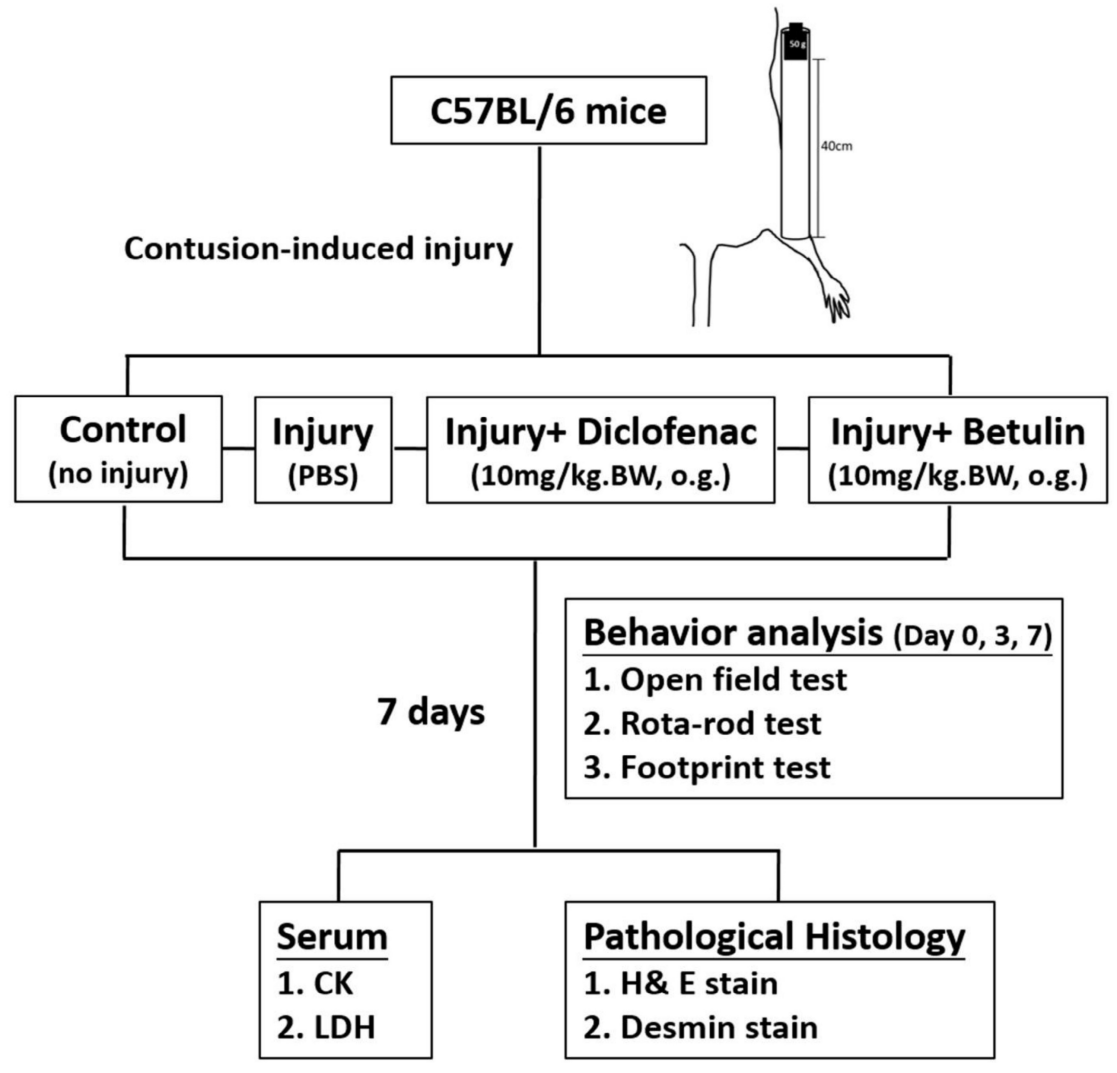
The flow chart of the study design after drop-mass-induced muscle injury in mice.

Drop-mass method was used to establish a muscle contusion model in the present study. Animals were randomly assigned into the following groups: 1. control group (no injury, PBS-treated), 2. injury group (with drop-mass injury, PBS-treated), 3. injury+ diclofenac group (with drop-mass injury, diclofenac-treated, 10 mg/kg body weight), and 4. injury+ betulin group (with drop-mass injury, betulin-treated, 10 mg/kg body weight) (Figure 1). Open field test and rota-rod test were performed to evaluate muscle function under spontaneous and constrained locomotor activities, respectively.

**Figure 2:**
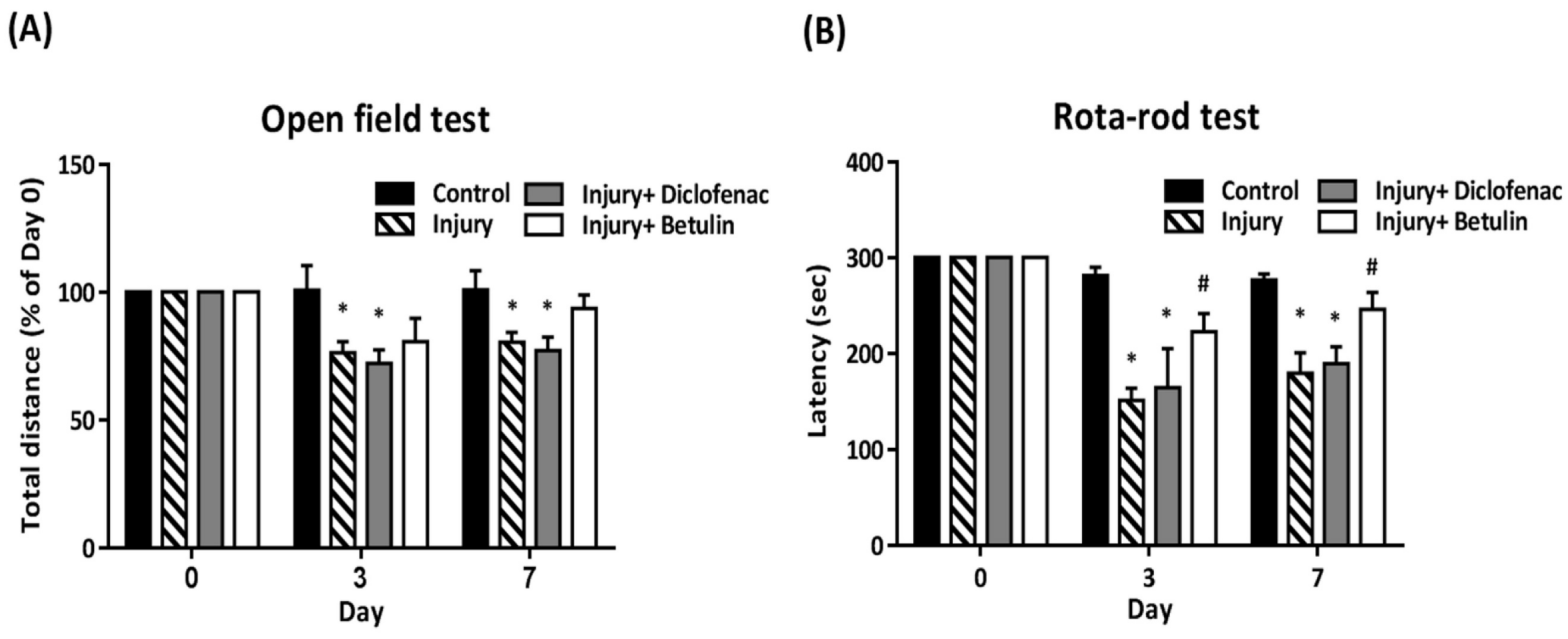
** Betulin treatment improves locomotor functions after drop-mass-induced muscle injury.** Animals were randomly assigned into the following groups: 1. control group (no injury, PBS-treated), 2. injury group (with drop-mass injury, PBS-treated), 3. injury+ diclofenac group (with drop-mass injury, diclofenac-treated), and 4. injury+ betulin group (with drop-mass injury, betulin-treated). (A) Open field test, and (B) Rota-rod test were performed on 0, 3, and 7 days after injury. Data were presented as mean ± standard error mean. * indicates a significant difference at *p* < 0.05 compared with the control group by one-way ANOVA. # indicated a significant difference at *p* < 0.05 compared with the injury group by one-way ANOVA.

In open field test, total distance (% of 0 day) was significantly decreased in the injury and injury+ diclofenac groups on 3 and 7 days after drop-mass-induced injury, but the decrease was not observed in the injury+ betulin group (Figure 2A). The rota-rod test was performed to assess motor performance after drop-mass-inudced muscle injury. The higher latency time means better motor ability. The data revealed that latency time was decreased after injury, especially in both injury and injury+ diclofenac groups (Figure 2B). Interestingly, latency time in the injury+ betulin group was significantly recovered as compared to injury group (Figure 2B).

**Figure 3:**
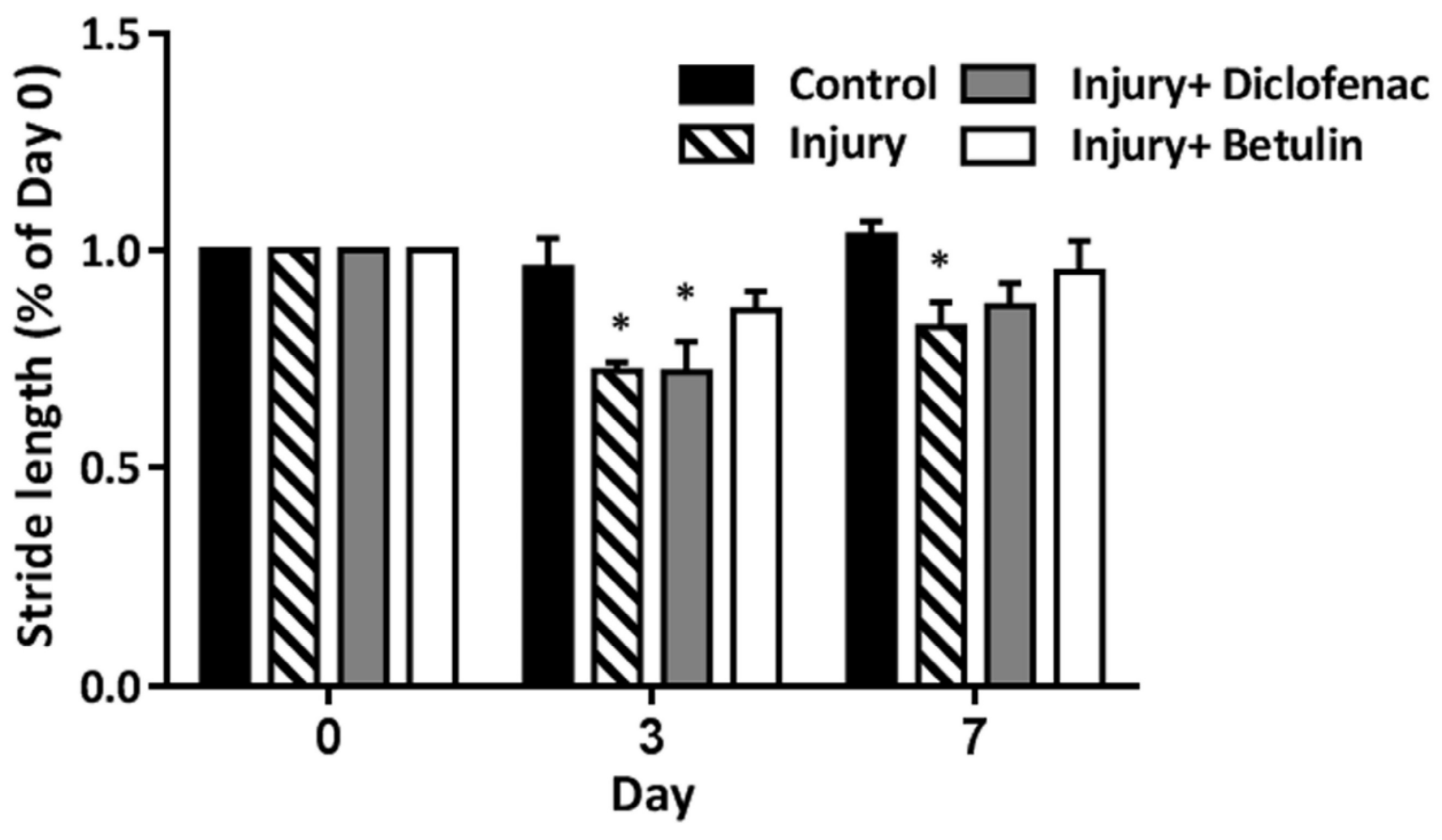
** Betulin treatment improves stride length on footprints after drop-mass-induced muscle injury.** Animals were randomly assigned into the following groups: 1. control group (no injury, PBS-treated), 2. injury group (with drop-mass injury, PBS-treated), 3. injury+ diclofenac group (with drop-mass injury, diclofenac-treated), and 4. injury+ betulin group (with drop-mass injury, betulin-treated). Footprint analysis was performed on 0, 3, and 7 days after injury. Then the stride length was measured between the second to fourth footprints of the damaged side. Data were presented as the mean ± standard error mean. * indicates a significant difference at *p* < 0.05 compared with the control group by one-way ANOVA.

Stride length is the distance between the continuous steps. The stride length become shorter when the mice have discomfort caused by muscle injury. On 3 days after drop-mass-induced muscle injury, the stride length was significantly shorted in both injury and injury+ diclofenac groups, but not in injury+ betulin group (Figure 3). It is of note that the stride length in injury group was still shorted on 7 days after injury. These findings indicated that betulin treatment improved motor functions.

### Betulin attenuates the biomarkers of muscle damage after drop-mass-induced muscle injury

**Figure 4:**
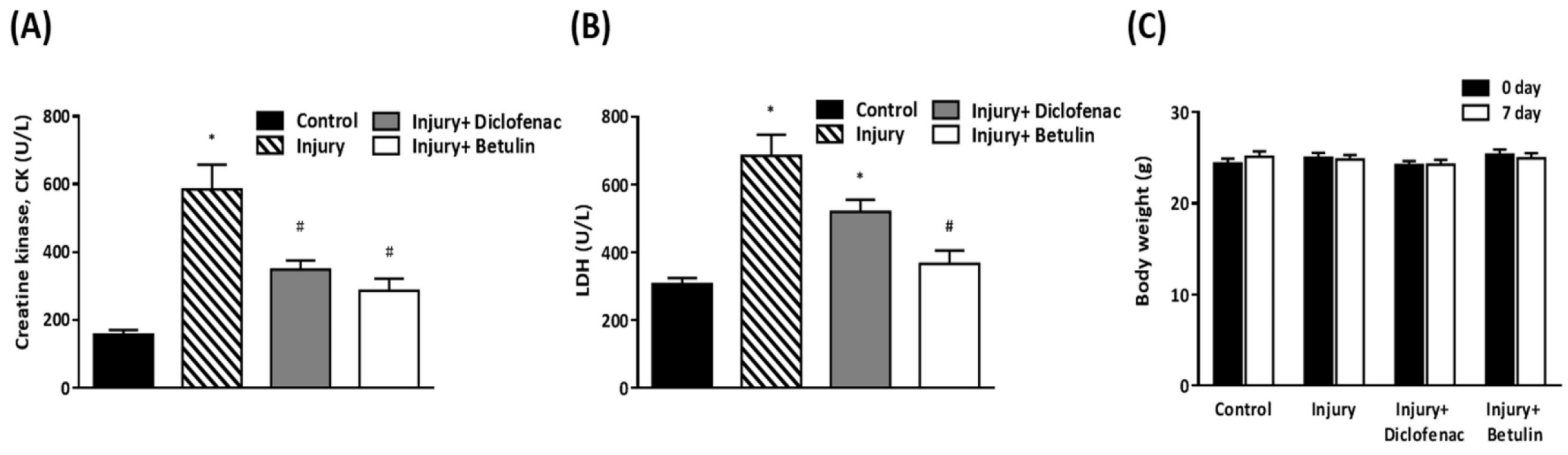
** Betulin treatment decreases serum creatine kinase (CK), lactate dehydrogenase (LDH) levels after drop-mass-induced muscle injury.** Animals were randomly assigned into the following groups: 1. control group (no injury, PBS-treated), 2. injury group (with drop-mass injury, PBS-treated), 3. injury+ diclofenac group (with drop-mass injury, diclofenac-treated), and 4. injury+ betulin group (with drop-mass injury, betulin-treated). (A) Serum CK levels and (B) Serum LDH levels on 7 days after drop-mass-induced muscle injury. (C) The changes of body weight during the experiment. Data were presented as the mean ± standard error mean. * indicates a significant difference at *p* < 0.05 compared with the control group by one-way ANOVA. # indicated a significant difference at *p* < 0.05 compared with the injury group by one-way ANOVA.

Serum creatine kinase (CK), and lactate dehydrogenase (LDH) are commonly used clinical indicators of muscle damage 21, 22. CK levels in injury group were significantly higher than control group (585±72.4 vs 157.4±13 U/L, *p* < 0.05) as shown in Figure 4A. Importantly, the CK levels were decreased by 40% and 50% in injury+ diclofenac and injury+ betulin groups, respectively, compared with the injury group. In addition, the LDH levels in the injured group were significantly higher than the control group (685.1±61.7 vs 307.2±17.2 U/L,* p* < 0.05) (Figure 4B). However, the LDH levels in injury+ diclofenac or injury+ betulin groups were significantly decreased (-25% and -50%, respectively) compared with the injury group. Thus, betulin treatment seems to ameliorate the muscle damage. It is noted that the LDH levels in injury+ betulin group were attenuated to reach basal levels almost. In addition, the body weight of animals was recorded. There was no significant change in body weight in these animals during the experiment (Figure 4C).

### Betulin alleviates inflammation and improves muscle regeneration

**Figure 5:**
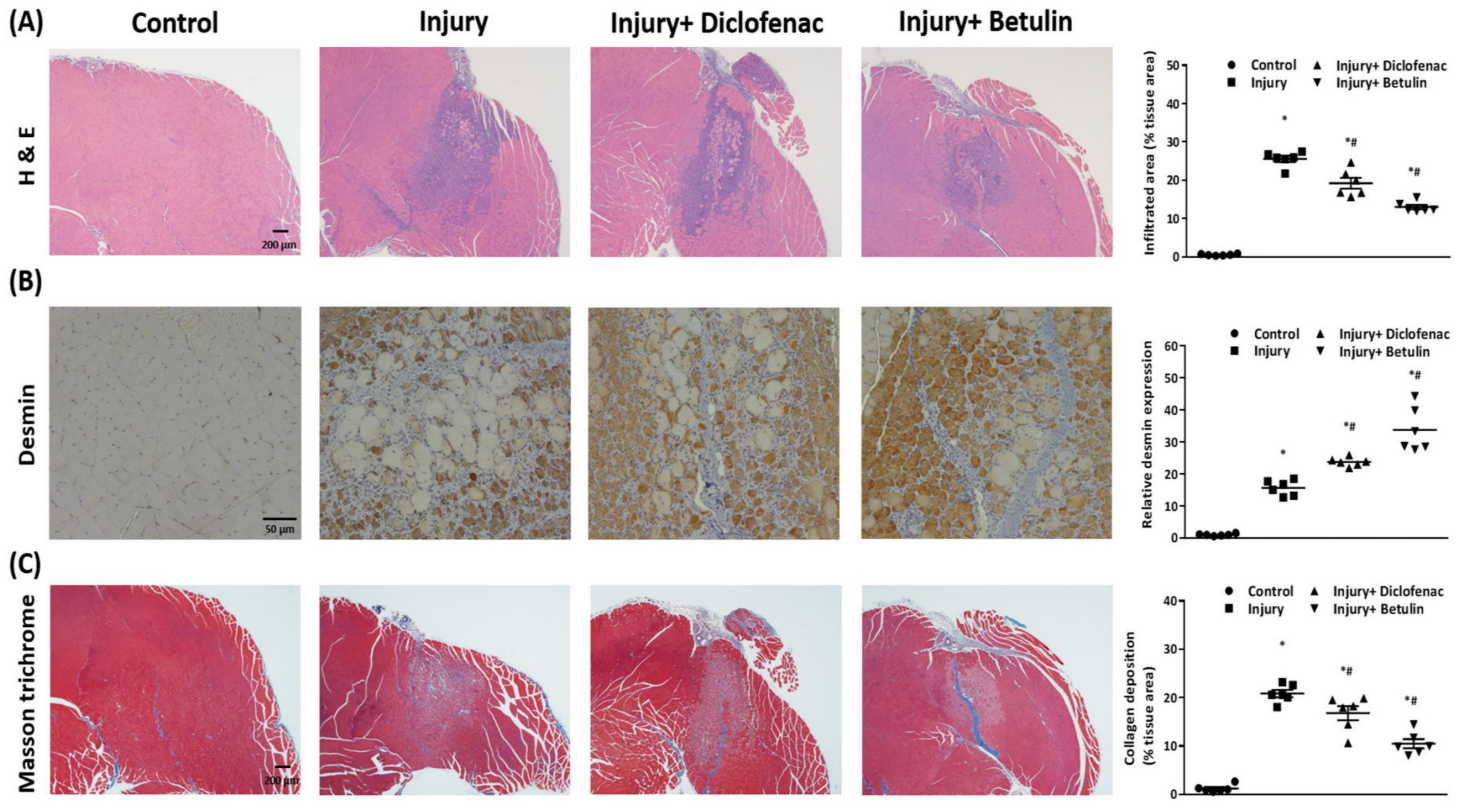
** Betulin treatment decreases neutrophil infiltration, raises protein levels of desmin, and alleviates deposition of collagen fibers in injured muscle tissues.** Muscle samples were observed by a bright field microscopy. Animals were randomly assigned into the following groups: 1. control group (no injury, PBS-treated), 2. injury group (with drop-mass injury, PBS-treated), 3. injury+ diclofenac group (with drop-mass injury, diclofenac-treated), and 4. injury+ betulin group (with drop-mass injury, betulin-treated). (A) H& E staining. Bar scale is 200 µm (magnification 40×). The infiltrated areas were quantified and showed. (B) Desmin staining. Bar scale is 50 µm (magnification 100×). The levels of desmin expression were quantified and showed. (C) Masson's trichrome staining of drop-mass-induced muscle injury, with collagen fibers stained blue and muscle fibers stained red. Bar scale is 200 µm (magnification 40×). The areas of collagen deposition were quantified and showed. * indicates a significant difference at *p* < 0.05 compared with the control group by one-way ANOVA. # indicated a significant difference at *p* < 0.05 compared with the injury group by one-way ANOVA.

Histopathology analysis were used to evaluate recovery of the injured muscle. We observed that the muscle cell was regularly arranged in the control group, but the disrupted muscle fibers and the neutrophil infiltration were found around the injury site in the injury group on 7 days after injury (Figure 5A). Interestingly, the inflammatory responses were mitigated and the regions of neutrophil infiltration were diminished after diclofenac or betulin treatment on 7 days after injury, especially in injury+ betulin group (Figure 5A). Desmin is a commonly used indicator to assess regeneration of muscle after injury. The data showed that the level of desmin was increased after injury, and it was higher both in injury+ diclofenac and injury+ betulin groups compared with injury group (Figure 5B). Masson's trichrome staining is used to highlight collagen fibers in injured muscle. Depositions of collagen fibers could impair muscle healing. As showed in Figure 5C, less collagen fibers were observed both in injury+ diclofenac and injury+ betulin groups compared with these in injury groups. Thus, we suggested that diclofenac or betulin could be effective treatments for muscle repair.

## Discussion

Muscle contusion can be caused by exercise or an impact by a mass, and it causes pain, swelling or impaired muscle functions 23. Conventional treatments include RICE (rest, ice, compression, and elevation) therapy along with nonsteroidal anti-inflammatory drugs (NSAIDs) to relieve discomfort. The present study exhibited betulin as a potential compound to treat muscle injury. The data showed that betulin treatment enhanced the recovery of motor functions in a mouse model of muscle contusion that induced by drop-mass method. Betulin treatment also attenuated serum creatine kinase (CK) and lactate dehydrogenase (LDH) levels after muscle injury, and decreased neutrophil infiltration and increased protein levels of desmin in the injured muscle tissues.

Serum creatine kinase (CK) exists in skeletal muscle, heart, and brain 21, and lactate dehydrogenase (LDH) is extensively expressed in body tissues. CK and LDH are the commonly used clinical indicators of muscle damage 21, 22. Higher CK and LDH levels and massive neutrophil infiltration are observed in animal models of muscle damage 22, 24-26. Olanlokun et al. indicate that betulinic acid, one oxidized form of betulin, decreases CK activity to attenuate malaria infection-induced cardiac and skeletal injury 27. Our data revealed that betulin treatment not only attenuated CK and LDH levels (Figure 4) but also blunted neutrophil infiltration in damaged sites (Figure 5A) after contusion-induced muscle injury. Thus, betulin may play an important role as an inflammatory modulator and to attenuate muscle damage during the repair process.

Several studies indicate that betulin regulates the inflammatory responses by inhibiting the release of pro-inflammatory cytokines 14, 15. However, less studies explore the effects of betulin on muscle synthesis. One study reveals that chickens fed with a betulin-based diet have higher body weight duo to muscle fiber rather than fat accumulation 28. George et al. reveal that protein levels of desmin (muscle regeneration marker) is increased to reach its highest level on 7 days after damage, and more abundant desmin protein may represent the better muscle recovery 8. In this study, histopathology analysis showed that betulin treatment increased the levels of desmin in the injured muscle on 7 days after contusion-induced muscle injury. In the same time, this treatment decreased neutrophil infiltration and alleviated deposition of collagen fibers as well. The formation of collagen fibers is beneficial for muscle healing, but overproduction of collagen fibers lead to scars and thus impairs the regeneration and functional recovery of muscle 29. Based on these findings, we suggested that betulin may enhance muscle regeneration, and thus recover motor functions.

Furthermore, betulin treatment could be effective to recover stride lengths in footprints analysis in injured mice (Figure 3). Previous reports show that shortened stride lengths are observed in collagen-induced arthritis rodent model and exercise-induced muscle injury model 30, 31. The authors consider this change may result from a hesitation to put weight on the injured limb. In our data, we also observed that the stride length was decreased after contusion-induced muscle injury (Figure 3). We suggested that this behavioral change may be associated with pain or discomfort after muscle injury. However, mice treated with betulin had normal stride length after injury. Betulin has been demonstrated to have excellent analgesic activity in models of acetic acid-induced writhing response and formalin test 32. Therefore, we suggested that betulin treatment may alleviate the pain or discomfort caused by injury, and thus be beneficial to recover stride lengths in injured mice.

Jäger et al, show that a triterpene extract from outer bark of birch consisting mainly of betulin is administered to rats (540 mg/kg, intraperitoneal injection for 28 days) 33**.** No toxic symptoms or no histopathological changes and mortality are observed in these rats. In our study, mice were treated with betulin (10 mg/kg, oral gavage for 7 days). There was no significant change in body weight in these animals. These findings suggest that betulin should be safe or low toxic, and is potential for further application.

## Conclusion

The present study revealed that betulin treatment after contusion-induced muscle injury attenuated muscle damage, improved muscle regeneration, and restored motor function. Thus, betulin could be developed into potential medicinal applications for treating contusion-induced muscle injury.
